# Angle closure caused by a plateau-like iris associated with an enlarged Soemmering’s ring: a case report

**DOI:** 10.1186/s12886-016-0226-0

**Published:** 2016-05-04

**Authors:** Fumika Kitamura, Toshihiro Inoue, Utako Kuroda, Hidenobu Tanihara

**Affiliations:** Department of Ophthalmology, Faculty of Life Sciences, Kumamoto University, 1-1-1, Honjo, Chuo-ku, Kumamoto Japan

**Keywords:** Angle closure glaucoma, Pilocarpine, Soemmering’s ring

## Abstract

**Background:**

This study evaluated the anterior ocular segment in a pseudophakic eye with angle closure due to a plateau-like iris associated with Soemmering’s ring, using ultrasound biomicroscopy (UBM) and anterior segment optical coherence tomography (AS-OCT).

**Case presentation:**

A 60-year-old woman was referred from a local clinic due to sudden-onset ocular pain and uncontrolled intraocular pressure (IOP) in the left eye, which was 56 mmHg after treatment with latanoprost, timolol, and dorzolamide eye drops. Fourteen years earlier, she developed acute primary angle closure. At that time, because the IOP remained elevated after a peripheral iridectomy, cataract extraction combined with goniosynechialysis was added. After the IOP decreased to within the normal range, a secondary intraocular lens was implanted outside the bag. On this admission, UBM and AS-OCT images showed angle closure caused by the combination of a plateau-like iris and contact between the mydriatic pupillary margin and enlarged Soemmering’s ring. After adding 2 % pilocarpine four times a day, the mydriasis resolved slightly, and the IOP decreased to the normal range between 8 and 18 mmHg. AS-OCT images showed re-opening of the angle structure after treatment with 2 % pilocarpine.

**Conclusion:**

The intraocular pressure and angle structure in eyes with a plateau iris after cataract extraction should be followed carefully.

## Background

Acute angle closure has various causes, including pupillary block, plateau iris, and secondary mechanisms (e.g., neovascularization, iris tumor, and aqueous misdirection) [1]. In pseudophakic eyes, some investigators have reported the occurrence of angle closure caused by Soemmering’s ring [2, 3]. Soemmering’s ring is a doughnut-like structure that results from the proliferation of lens epithelial cells in the peripheral part of the capsular bag remaining after cataract extraction. However, the causative mechanism of angle closure differs from case to case. Kobayashi et al. reported a case of pupillary block related to enlargement of Soemmering’s ring, in which a laser iridotomy overcame the pupillary block, reducing the intraocular pressure (IOP) [2]. Kung et al. reported a non-pupillary block mechanism in which progressive synechial angle closure was induced by enlargement of Soemmering’s ring [3]. Here, we report another non-pupillary block mechanism, involving a combination of a plateau-like iris and enlarged Soemmering’s ring in an eye with a mydriatic pupil.

## Case presentation

A 60-year-old woman was referred from a local clinic with sudden-onset ocular pain and uncontrolled IOP in the left eye, which was 56 mmHg after treatment with latanoprost, timolol, and dorzolamide eye drops. Fourteen years earlier, she developed acute primary angle closure in both eyes. At that time, a peripheral iridectomy was performed in both eyes to relieve the pupillary block, resulting in controlled IOP levels in the right eye, but not in the left. Subsequently, cataract extraction combined with goniosynechialysis (GSL) was performed in the left eye and the IOP levels normalized. Secondary implantation of an intraocular lens (IOL) was performed, resulting in out-of-the-bag fixation of the implanted IOL. The detail of the cataract surgery is unknown, because the medical record is not available.

**Fig. 1:**
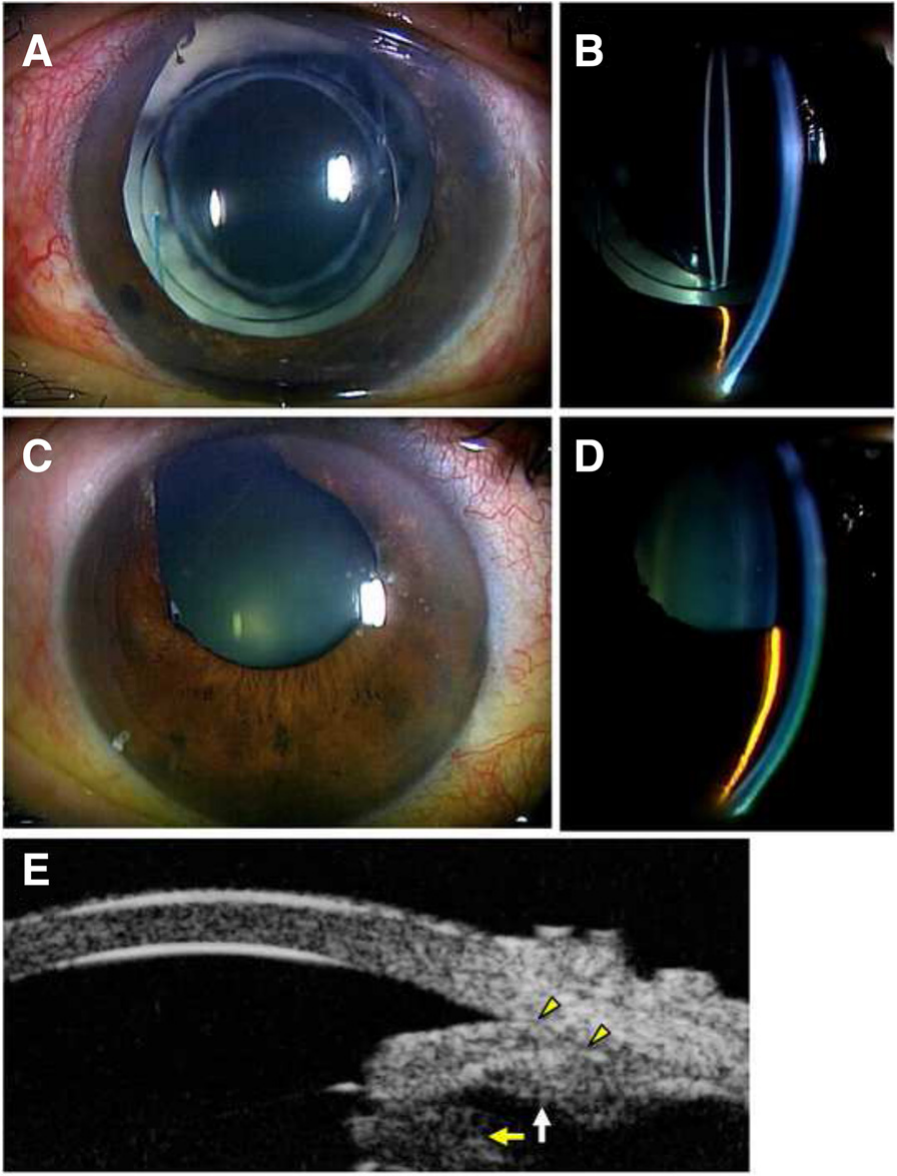
Slit lamp photographs. In the *left* eye, the pupil was markedly dilated, and a patent peripheral iridectomy and Soemmering’s ring were found (**a**, **b**). In the *right* eye, the anterior chamber was shallow despite a patent iridectomy. In the left eye, the anterior chamber was deep in the central region, but very shallow in the periphery, with a plateau-like iris configuration (**c**, **d**). Ultrasound biomicroscopy (UBM) showed a plateau-like iris and co-localized dilated pupil margin (between *yellow arrow heads*) with an enlarged Soemmering’s ring (*yellow arrow*) and anterior insertion of ciliary process (*white arrow*) (**e**)

**Fig. 2:**
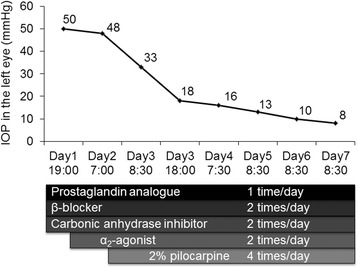
Intraocular pressure (IOP) changes and medical treatments

**Fig. 3:**
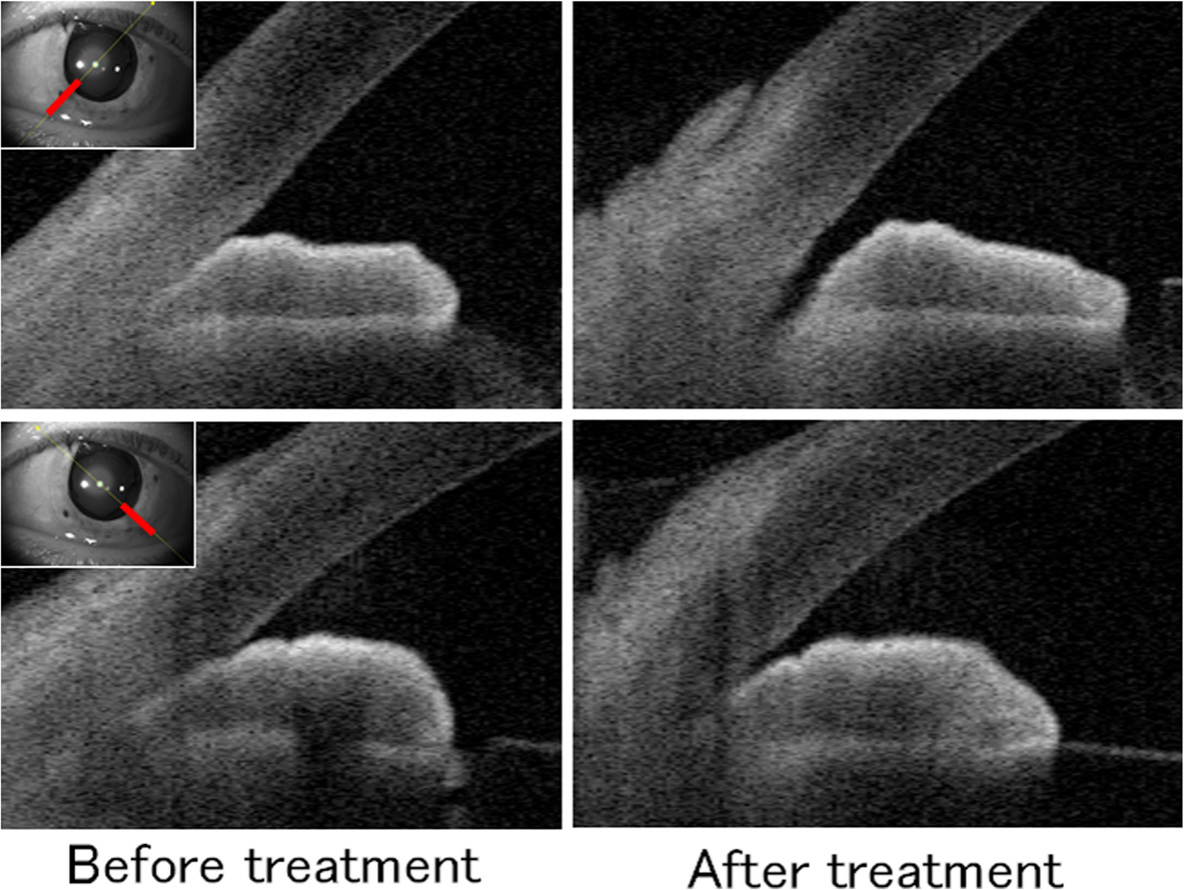
Anterior segment optical coherence tomography (AS-OCT) and UBM images. AS-OCT showed re-opening of the angle structure after medical treatment. The insets correspond to slices at the red lines

At the first examination in our department, slit lamp examination of the left eye showed corneal edema, a markedly dilated pupil, a patent iris coloboma created by the previous peripheral iridectomy, and the presence of Soemmering’s ring in the peripheral part of the remaining capsular bag (Fig. 1a, b). The anterior chamber of the right eye was somewhat shallow due to anterior positioning of the crystalline lens (Fig. 1c, d), while in the left eye, it was deep in the central region, but very shallow in the periphery (Fig. 1b). Ultrasound biomicroscopy (UBM) showed that the iris had been displaced anteriorly by the enlarged Soemmering’s ring (Fig. 1e). The IOP was 8 mm Hg in the right eye and 50 mm Hg in the left (Fig. 2). Gonioscopic observation showed a narrow angle (Shaffer grade 1) in the right eye, and total closure of the angle structure in the left eye. Anterior segment optical coherence tomography (AS-OCT) showed a short, thick iris with a plateau-like iris configuration and angle closure due to the crowded angle structure in the left eye (Fig. 3). Despite treatment with a carbonic anhydrase inhibitor, β-blocker, prostaglandin analogue, and α_2_-agonist eye drops, the IOP remained elevated at 48–50 mmHg. After adding 2 % pilocarpine four times a day, the mydriasis resolved slightly, and IOP decreased to 8–18 mmHg, in the normal range (Fig. 2). AS-OCT images showed re-opening of the angle structure after treatment with 2 % pilocarpine.

Primary angle closure can be induced by pupillary block or a plateau iris (non-pupillary block). In our case, the angle closure did not result from a pupillary block mechanism, due to the patent (surgical) peripheral iridectomy and flat iris seen on AS-OCT and UBM. In addition, the UBM image showed a plateau-like iris and a crowded angle structure in the left eye. Clinically, the IOP can increase after the relief of pupillary block mechanisms in some eyes with narrow or closed angles, especially in Asians [4]. However, residual angle closure can be inhibited by lens extraction, as shown by UBM [5]. In some cases, iridociliary apposition in a plateau iris can persist after cataract extraction [6]. Even with a patent laser peripheral iridotomy, which relieves the pupillary block, about 30 % of the eyes with primary angle closure glaucoma had a plateau iris on UBM in Asian patients [7]. Furthermore, a secondary plateau iris configuration is often found after relief of synechial closure by GSL, although it is difficult to differentiate a surgically induced plateau iris configuration from a plateau iris that existed before the angle closure developed [8]. In our case, UBM demonstrated the anterior insertion of ciliary processes in the left eye (Fig. 1e) and a shallow anterior chamber in the right eye (data not shown), suggesting a pre-existing plateau-like iris before GSL. There was no history or UBM finding of secondary angle closure mechanisms, such as neovascularization, uveitis, iridociliary tumor, a dislocated IOL, iridovitreal block, or aqueous misdirection (malignant glaucoma). Therefore, it is quite likely that the plateau-like iris contributed to the episode of acute angle closure, although 14 years had passed since the GSL and cataract extraction. Therefore, additional factors may have contributed to the angle closure in our case. An enlarged Soemmering’s ring was noted in contact with the posterior surface of the iris in the dilated pupil margin, resulting in anterior displacement of the iris plane. It is possible that anterior swelling of Soemmering’s ring pushed the iris tissue causing deterioration of the plateau-like iris and closing the angle structure. This hypothesis is supported by the fact that the angle re-opened and the IOP fell after treatment with pilocarpine eye drops.

In conclusion, our case suggests that swelling of Soemmering’s ring can contribute to angle closure in eyes with a plateau-like iris. To our knowledge, this is the first report in which enlargement of Soemmering’s ring contributed to angle closure in addition to non-pupillary block mechanisms (plateau iris).

## Conclusion

The intraocular pressure and angle structure in eyes with a plateau iris configuration after cataract extraction should be followed carefully.

### Ethics and consent to participate

Not Applicable.

### Consent to publish

Written informed consent was obtained from the patient for publication of this case report and accompanying images. A copy of the consent is available for review by the Editor of this journal. All procedures conformed to the Declaration of Helsinki.

### Availability of data and materials

All the data supporting our findings is contained within the manuscript.

## Abbreviations

AS-OCT: anterior segment optical coherence tomography
GSL: goniosynechialysis
IOL: intraocular lens
IOP: intraocular pressure
UBM: ultrasound biomicroscopy
